# Nanostructured Strategies for Melanoma Treatment—Part II: Targeted Topical Delivery of Curcumin via Poloxamer-Based Thermosensitive Hydrogels

**DOI:** 10.3390/ph18030337

**Published:** 2025-02-27

**Authors:** Valentina Paganini, Daniela Monti, Patrizia Chetoni, Susi Burgalassi, Andrea Cesari, Fabio Bellina, Silvia Tampucci

**Affiliations:** 1 Department of Pharmacy, University of Pisa, Via Bonanno 33, 56126 Pisa, Italy; valentina.paganini@phd.unipi.it (V.P.); patrizia.chetoni@unipi.it (P.C.); susi.burgalassi@unipi.it (S.B.); silvia.tampucci@unipi.it (S.T.); 2 Italian Inter-University Center for the Promotion of the 3Rs in Teaching and Research, University of Pisa, 56122 Pisa, Italy; 3 Department of Chemistry and industrial Chemistry, University of Pisa, 56124 Pisa, Italy; andrea.cesari@unipi.it (A.C.); fabio.bellina@unipi.it (F.B.)

**Keywords:** curcumin, thermosensitive hydrogel, nanocarrier, topical melanoma therapy, skin delivery, polymeric micelles, sol–gel transition, confocal microscopy

## Abstract

**Background/Objectives**: Curcumin (CUR) is a natural compound with notable antitumor properties but faces limitations in topical applications due to poor aqueous solubility, instability, and insufficient skin penetration. To overcome these challenges, a nanomicellar formulation (TPGS30ELP15) was developed to enhance CUR solubility, stability, and skin penetration. This study aimed at evaluating the skin permeation and retention of CUR when delivered through nanomicelles alone or combined with a thermosensitive hydrogel for potential melanoma therapy. **Methods**: A CUR-loaded nanomicellar formulation containing CUR 5 mM was developed, characterized by particle sizes of 12–25 nm. Skin permeation studies utilized pig ear skin to assess CUR localization using both HPLC quantitative analysis and confocal microscopy. To improve patient comfort and application efficiency, the nanomicellar dispersion was incorporated into a thermosensitive hydrogel based on 16% Kolliphor^®^ P407 and was able to undergo a sol–gel transition at skin temperature (32–36 °C). Formulations were evaluated for physicochemical properties, stability, and CUR distribution within skin layers using in vitro permeation assays. **Results**: CUR-loaded nanomicelles demonstrated selective localization in the viable epidermis (100–150 µm depth), bypassing the stratum corneum. The addition of the thermosensitive hydrogel enhanced CUR retention and distribution, prolonging contact at the application site and providing a gradual release profile. The hydrogel’s sol–gel transition properties can facilitate ease of use and patient compliance. The combined system effectively delivered CUR to the basal epidermis, a target site for melanoma treatment, achieving therapeutically relevant drug concentrations. **Conclusions**: The incorporation of CUR-loaded nanomicelles into a thermosensitive hydrogel enhanced the solubility, stability, and targeted delivery of CUR to skin layers. This dual system represents a promising strategy for improving topical drug delivery for melanoma therapy, addressing limitations associated with CUR’s physicochemical properties while ensuring patient-friendly application and gradual drug release.

## 1. Introduction

In a previous study, a nanostructured formulation was developed to enhance the topical delivery of CUR for melanoma treatment, addressing its inherent limitations such as poor aqueous solubility, instability in aqueous environments, and susceptibility to degradation upon light exposure [1]. CUR is recognized for its diverse biological activities, particularly its antitumor effects, which include the inhibition of cell proliferation, the suppression of inflammatory mediators, such as interleukins and tumor necrosis factor, and the modulation of various cell signaling pathways [2,3]. However, its therapeutic potential is constrained by its lipophilicity and chemical instability, which significantly reduces its effectiveness in topical applications [4,5]. To overcome these challenges, the nanostructured system was designed to increase CUR’s aqueous solubility, prolong its stability, and protect its molecular integrity from environmental stressors such as oxidative degradation, high temperatures, and pH [6]. This formulation successfully improved CUR solubility several-fold, prolonged its stability, and demonstrated efficacy in reducing the viability of melanoma cells (A375) while preserving safety in fibroblasts [1].

The current study aimed at evaluating the cutaneous permeation/penetration of CUR from the selected micellar dispersion applied directly to the skin or after being introduced into a thermosensitive hydrogel. The skin, with its complex barrier properties, especially the stratum corneum (SC), presents considerable challenges for drug penetration [7]. Nanostructured delivery systems, however, offer significant advantages by improving drug localization and penetration without invasive methods. Nanocarriers enhance bioavailability and target site selectivity by interacting with skin lipids and penetrating hair follicles, forming depots for sustained release. This mechanism facilitates CUR delivery to deeper skin layers while bypassing the SC [8].

The physicochemical properties of nanocarriers (e.g., size, shape, charge, and stiffness) are crucial in determining their interaction with biological systems and their ability to penetrate the skin layers. Nanostructures can pass through the skin intact, fuse with the skin surface, or follow the follicular pathway. The literature on nanostructured systems for skin application highlights their ability to penetrate the skin and, in many cases, disrupt the skin barrier to improve drug penetration or retention by directing the drug to the basal layer of the epidermis, a key site for the treatment of diseases, such as melanoma [9]. To enhance patient comfort, improve drug delivery efficiency, and minimize leakage from the application site, the micellar formulation was incorporated into a thermosensitive hydrogel [10,11]. Such hydrogels gel rapidly upon contact with skin at physiological temperatures, eliminating the need for organic solvents, cross-linking agents, or external equipment. This sol–gel transition facilitates localized retention of the drug, achieving higher concentrations at the target site while mitigating systemic toxicity [12].

Kolliphor^®^ P407 (poloxamer 407) was chosen as polymeric material for its well-documented sol–gel transition properties, with the gelation temperature decreasing as the concentration increases [13]. Poloxamers, marketed under various trade names, such as Pluronic^®^, Synperonic^®^, and Lutrol^®^, are triblock copolymers composed of a central hydrophobic polypropylene oxide (PPO) block flanked by two hydrophilic polyethylene oxide (PEO) blocks [14,15]. This unique structure imparts remarkable amphiphilic and thermosensitive characteristics, making poloxamers ideal for pharmaceutical applications. Notably, poloxamer 407 is recognized by the US Food and Drug Administration (FDA) as an “inactive ingredient” suitable for various human formulations [16,17,18].

Aqueous dispersions of poloxamer 407 exhibit thermoreversible gelation behavior, characterized by a sol–gel transition temperature (T_sol–gel_). Below T_sol–gel_, the poloxamer 407 aqueous solutions remain fluid, while above this temperature, hydrophobic interactions between the polymeric chains occur, and the copolymer chains aggregate into micelles. Micellization is a result of the dehydration of the hydrophobic PPO blocks [13,14,17]. Upon reaching a specific temperature, the micelles come into contact and organize into a network structure, leading to the formation of a hydrogel, as indicated by a marked increase in viscosity [10].

In this study, an optimized concentration of poly(ethylene glycol)-block-poly(propylene glycol)-block-poly(ethylene glycol) (Kolliphor^®^ P407) was selected to achieve a sol–gel transition range ideal for skin applications (32–36 °C). This thermosensitive hydrogel was loaded with CUR-containing nanomicelles to combine the advantages of enhanced drug stability, targeted delivery, and patient-friendly application. The final formulation was thoroughly characterized to assess its physicochemical properties and subjected to in vitro permeation and distribution studies using pig ear skin, demonstrating its potential for effective topical melanoma therapy.

## 2. Results and Discussion

### 2.1. CUR Nanomicelle-Loaded Thermosensitive Hydrogel (TPGS30ELP15-HY)

A previous formulation study [1] identified a nanostructured delivery system capable of solubilizing and stabilizing curcumin (CUR) in an aqueous medium, demonstrating time-dependent cytotoxicity against A375 melanoma cells. The therapeutic efficacy and safety of this nanomicellar system are dependent on particle size and encapsulation efficiency, as these parameters influence drug bioavailability and stability.

The nanomicellar formulation (TPGS30ELP15) incorporated CUR within a binary mixture of two non-ionic surfactants: Vitamin E TPGS (TPGS, d-α-Tocopherol Polyethylene Glycol Succinate) and Kolliphor ELP (ELP, Polyoxyl-35-castor Oil) in a 30:15 molar ratio. This formulation effectively solubilized CUR at a concentration of 5.51 ± 1.09 mM, with an entrapment efficiency of 67.72 ± 13.46%. The nanomicelles exhibited an aerodynamic diameter of 13.11 ± 0.01 nm and a polydispersity index of 0.371 ± 0.05, ensuring a well-defined nanoscale size distribution that is crucial for dermal penetration and sustained drug release. To enhance formulation stability and enable topical application, the nanomicellar system underwent freeze drying, yielding a lyophilized product (TPGS30ELP15-F) that was subsequently reconstituted in a 16% aqueous dispersion of Kolliphor^®^ P407 to form a thermosensitive hydrogel loaded with CUR nanomicelles (TPGS30ELP15-HY). Key parameters, including particle size, solubilized CUR content, and encapsulation efficiency, were systematically monitored to assess the impact of various experimental preparation steps on the integrity and performance of the final formulation.

The results are summarized in Table 1.

The nanomicellar size and the amount of encapsulated CUR remained consistent throughout the preparation steps. In the TPGS30ELP15-F and TPGS30ELP15-HY formulations, a decrease in PDI values was observed, indicating a more uniform size distribution after the freeze-drying process.

The reconstitution step of the freeze-dried product with the 16% Kolliphor^®^ P407 dispersion did not appear to induce any changes in the analyzed parameters, further demonstrating the hydrogel’s remarkable capacity to retain the encapsulated drug.

**Figure 1:**
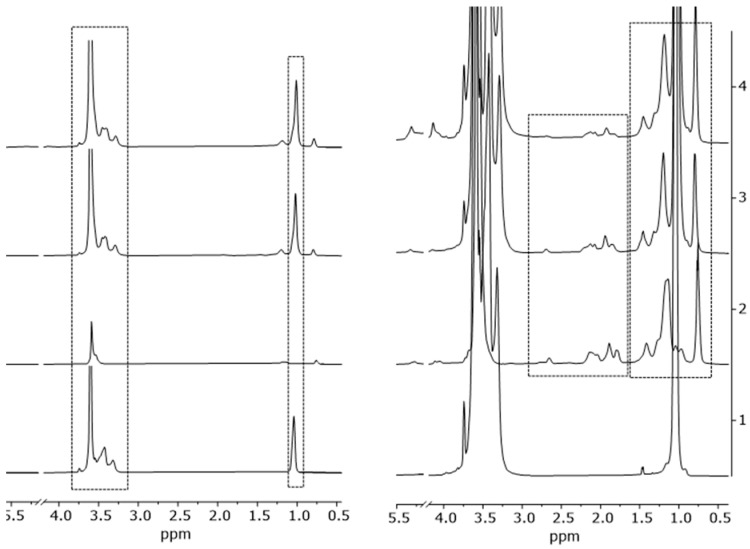
^1^H NMR (500 MHz, H_2_O + 5%D_2_O, 25 °C) spectra of (1) Lutrol F127, (2) TPGS30ELP15-empty, (3) TPGS30ELP15-HY-empty, (4) TPGS30ELP15-HY.

To assess whether the nanomicelles, either empty or CUR loaded, maintained their structural integrity in the presence of Kolliphor^®^ P407, Nuclear Magnetic Resonance (NMR) spectroscopic analysis was performed. Through NMR spectroscopy, it is possible to obtain a pool of detailed information on structure, composition, and interactions on nanogel systems [19] and heterogeneous assembly [20]. As a starting point, ^1^H NMR spectra of pure Kolliphor^®^ P407, empty nanomicelles (TPGS30ELP15-empty), empty nanomicelles in the presence of Kolliphor^®^ P407 (TPGS30ELP15-HY-empty), and CUR-loaded nanomicelles in the presence of Kolliphor^®^ P407 (TPGS30ELP15-HY) were recorded (Figure 1). In order to minimize sample treatment, the NMR tubes for analysis were prepared starting from water solutions, and they were then added to 5% of D_2_O for a spectrometer lock. No significant variations were observed in terms of peak chemical shifts and line shape, neither on Kolliphor^®^ P407 nor on nanomicelles, regarding TPGS30ELP15-HY. This demonstrates that Kolliphor^®^ P407 acts only as a gelled support without establishing strong disruptive interactions towards nanomicelles, which in turn keep their native structure.

**Figure 2:**
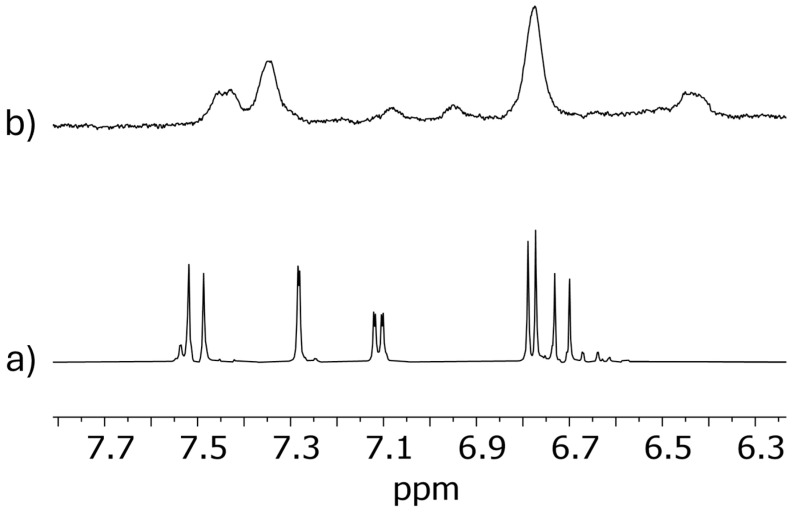
^1^H NMR (500 MHz, H_2_O+5%D_2_O, 25 °C) spectra in quantitative conditions of: (**a**) CUR in DMSO-d_6_ (5 mM) and (**b**) TPGS30ELP15-HY in H_2_O + 5% D_2_O.

In addition, it was also possible to confirm the presence of CUR in the TPGS30ELP15-HY sample (Figure 2). The evident line broadening of CUR signals can be attributed to the interaction with nanomicelles, as already observed [1].

**Figure 3:**
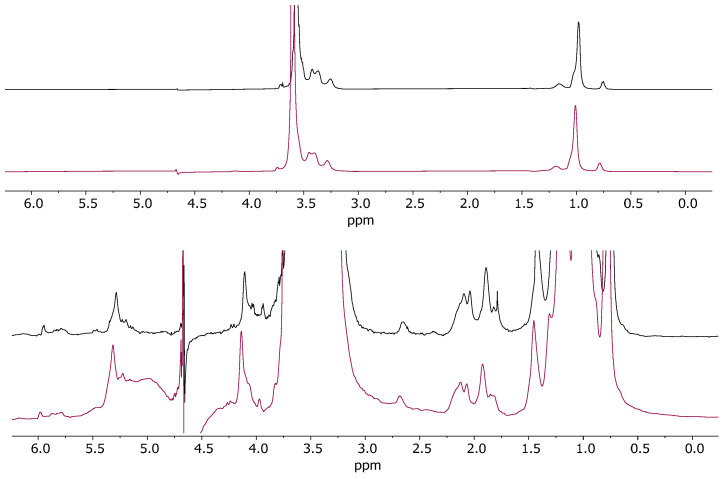
^1^H NMR spectra (500 MHz, H_2_O + 5%D_2_O, 25 °C) of TPGS30ELP15-HY at t_0_ (violet line) and after 4 months (black line).

Moreover, the stability of nanomicelles in the presence of Kolliphor^®^ P407 was assessed by ^1^H NMR spectra recorded at t_0_ and after 4 months (Figure 3). No significant variations in the chemical shift or lineshape of both Kolliphor^®^ P407 and nanomicelle resonances were observed, demonstrating the stability of the formulation in solution over a long period of time.

**Figure 4:**
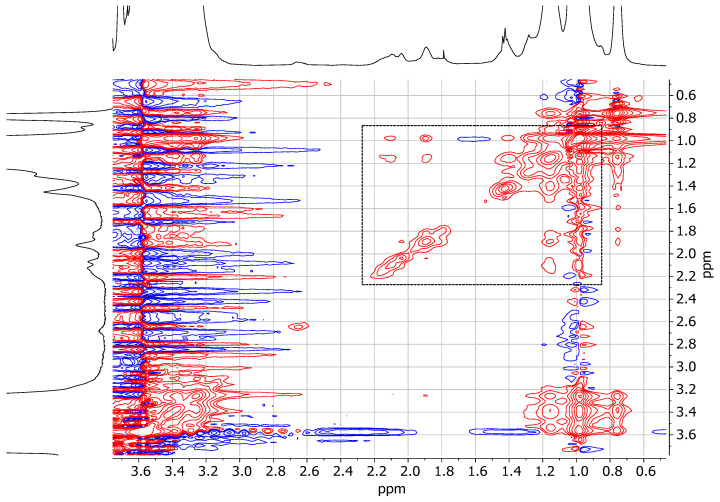
A 2D NOESY map (500 MHz, H_2_O + 5%D_2_O, 25 °C) spectra of TPGS30ELP15-HY.

Finally, a 2D NOESY map (Figure 4) was recorded for TPGS30ELP15-HY. By looking at the NOE cross-correlations, it is possible to see interesting effects between methyl moieties of Kolliphor^®^ P407 (~1.0 ppm) and the resonances of nanomicelles free from polymer supplementation (1.4–2.2 ppm). This demonstrates that a relevant percentage of nanomicelles are actually included in Kolliphor^®^ P407 gel. Nanomicelles appear to be located in the more hydrophobic shell of the gel rich in methyl fragments [21].

The nanostructured formulation was incorporated into a thermosensitive hydrogel due to its well-established advantages in drug delivery [12]. Temperature-responsive hydrogels undergo a phase transition upon exposure to physiological temperatures, shifting from a liquid state at room temperature to a gel at the application site. This transition allows for localized drug retention, thereby increasing drug concentration at the target site while reducing systemic exposure and minimizing potential toxicity and adverse effects [10].

A key advantage of this system is the immediate sol–gel transition upon reaching body temperature, occurring without the need for chemical initiators or enzymatic activation. This intrinsic responsiveness simplifies the formulation process, making it milder, more efficient, and cost effective. As a result, thermosensitive hydrogels represent one of the most straightforward and practical strategies for localized drug delivery, offering enhanced therapeutic efficacy with improved patient compliance [22,23].

**Figure 5:**
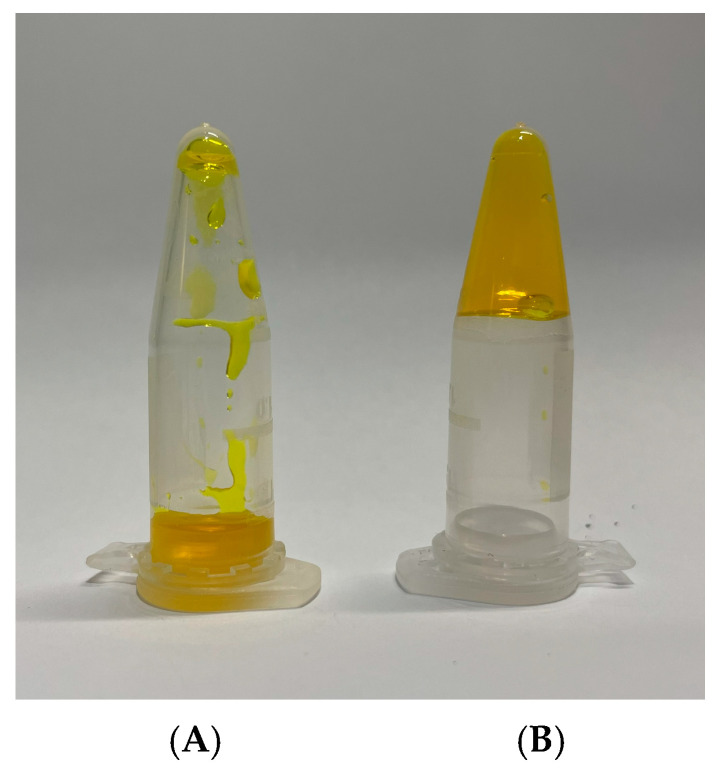
Sol-to-gel transition behavior of TPGS30ELP15-HY: (**A**) sol and (**B**) gel.

In the current study, the TPGS30ELP15-HY hydrogel was evaluated for its sol–gel transition temperature, a key property that enables the transformation of the easy-to-administer liquid colloidal dispersion into a gel upon contact with the skin surface at 32 °C, ensuring prolonged in situ retention of the formulation. The gelation transition was determined by the tube inversion method. This technique has previously been employed by various research groups to ascertain the gel boundary in gel–sol behavior. It is noteworthy that Kolliphor^®^ P407 aqueous dispersions undergo a thermoreversible sol–gel transition arising from the formation of micelles with a PPO core and PEO shell and the volume change of micelles due to the behavior of PEO/water and the lower critical solution temperature (LCST) of PPO/water [24]. Previous studies demonstrated that the gelation temperature decreases proportionally with increasing concentrations of Kolliphor^®^ P407. Sol–gel transitions within the temperature range of 32–36 °C, ideal for cutaneous application, were observed at polymer concentrations of approximately 15–16% *w*/*w*. A 16% *w*/*w* concentration of Kolliphor^®^ P407 was selected to prepare a thermosensitive hydrogel containing CUR-loaded nanomicelles. The nanomicelle-loaded thermosensitive hydrogel (TPGS30ELP15-HY) appeared as a clear, slightly viscous liquid; the results obtained with the tube inversion method highlighted that TPGS30ELP15-HY was capable of undergoing sol–gel transition at a temperature of 32 °C, when changes in appearance and consistency of the hydrogel were observed. Anyway, the system transparency remained even in the gel state, as shown in Figure 5.

**Figure 6:**
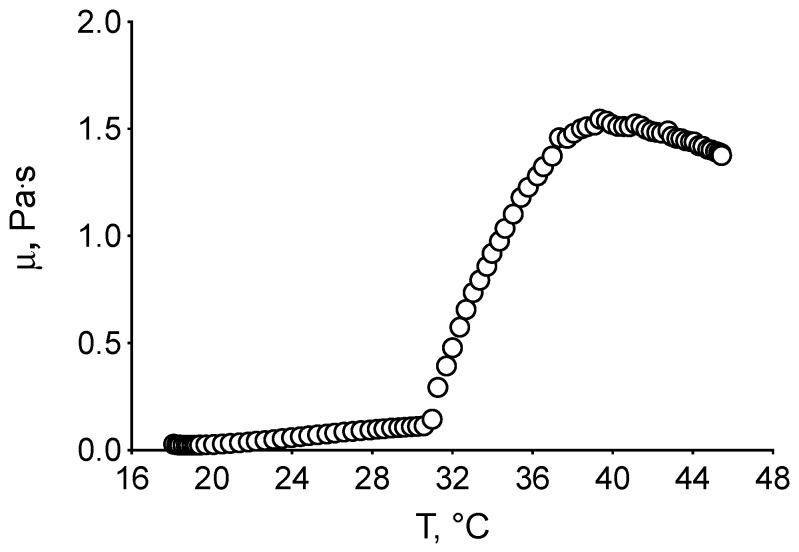
Flow curve of TPGS30ELP15-HY generated for temperature values ranging from 20 to 45 °C at a constant speed of 100 s^−1^ in 1000 s.

This behavior was confirmed by rheological analysis in rotational mode. Indeed, a sharp and abrupt increase in viscosity was observed when a 32 °C temperature was reached (Figure 6), demonstrating that TPGS30ELP15-HY behaves as a thermosensitive gel.

Furthermore, since it is generally believed that the phase transition occurs at the temperature where the storage modulus (G′) equals the loss modulus (G″) (cross-over temperature), the rheological behavior of the aqueous dispersions was further analyzed by frequency sweep. The mechanical behavior of conventional gel structure should show G′ always greater than G′′, while other combinations of these parameters define liquid or intermediate systems where entanglement between polymer chains might exist [25].

**Figure 7:**
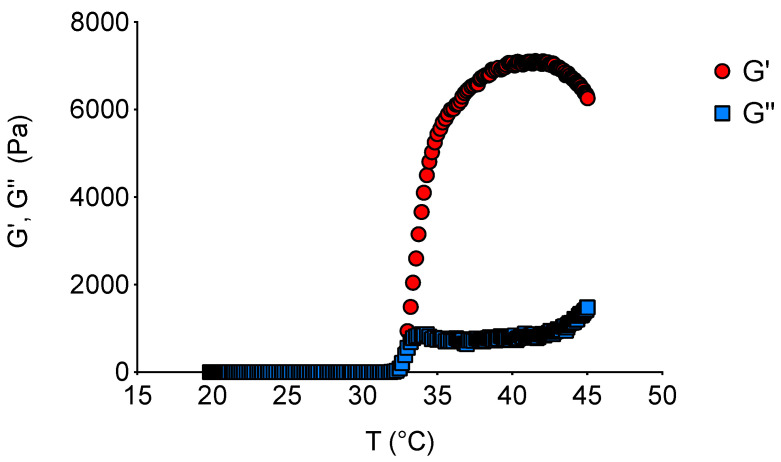
Effect of temperature on storage (G′) and loss (G″) moduli during the temperature sweep test.

As shown in Figure 7, G′ and G″ were recorded in the temperature range of 20–45 °C to highlight the sol–gel transition. At low temperatures, the TPGS30ELP15-HY behavior is liquid like (G″ > G′), transforming into a gel upon heating (G″ < G′), which is in accordance with other studies on sol–gel transitions [26]. The cross-over temperature for TPGS30ELP15-HY was found to be 32.78 °C, with G′ equal to G″ and measuring 334.9 Pa.

### 2.2. In Vitro CUR Release Studies

Table 2 summarizes the release parameters, including the release rate over 48 h and the amount of CUR released at 48 and 72 h. Both the TPGS30ELP15 nanomicellar dispersion and the TPGS30ELP15-HY hydrogel successfully released CUR throughout the 72 h monitoring period.

The release rate of CUR from the nanomicellar dispersion was 4.80 μg/h, higher than that of the thermosensitive hydrogel (TPGS30ELP15-HY), which was 2.09 μg/h with statistically significant differences. However, it can be observed that the nanomicellar dispersion released the maximum amount of CUR (11.38%) within the first 48 h of the experiment, with no further increase up to 72 h (11.12%). In contrast, the hydrogel formulation demonstrated a slower release, with CUR amounts increasing from 5.81% at 48 h to 7.25% at 72 h.

This pattern aligns with its structural characteristics and the gradual diffusion of CUR through the hydrogel matrix. Due to known stability issues of CUR, extending the experimental timeframe was not feasible; however, the release dynamics observed are consistent with those reported by Duan et al. [27], who described similar trends for CUR-loaded nanomicelles and gel formulations, showing release levels of approximately 40% and 15%, respectively.

Duan et al. [27] identified three primary factors contributing to the slow release of drugs from nanomicelles: (1) the stability of micelles formed by carrier materials with a low critical micelle concentration (CMC), which resist disassembly in aqueous environments; (2) the tight encapsulation of CUR within the micelle’s hydrophobic core; and (3) the high hydrophobicity of CUR, which slows its dissolution into the aqueous phase.

These mechanisms are also likely applicable to the findings of the present study. In the case of the hydrogel formulation (TPGS30ELP15-HY), the slower release rate can be attributed to an additional structural barrier provided by the polymeric network formed by Kolliphor^®^ P407 within the hydrogel matrix.

Before reaching the aqueous receptor phase, CUR must diffuse through both the micellar shell and the hydrogel matrix, significantly slowing its release rate. This dual-barrier effect is beneficial for topical applications, as it mitigates the risk of excessive CUR concentrations that could induce irritation or cytotoxicity while maintaining therapeutic levels at the target site for extended periods. Such release profiles are crucial for reducing the frequency of application and improving patient compliance in clinical settings.

### 2.3. Skin Permeation/Penetration Study

Skin permeation and penetration studies through pig ear skin were carried out on the TPGS30ELP15 nanomicellar dispersion and the hydrogel incorporating CUR-loaded nanomicelles. Testing the aqueous suspension of CUR under the same conditions as the control was not feasible due to the well-documented instability of CUR in an aqueous environment. This study aimed, first, to investigate the nanomicellar dispersion as a drug delivery system to optimize selective drug distribution within the target skin layer. Furthermore, it sought to evaluate the role of the thermosensitive hydrogel (TPGS30ELP15-HY) in establishing intimate contact with the skin surface, thereby enhancing the retention of active compounds within the tissue.

Pig ear skin was chosen because it is widely acknowledged as an excellent model for human skin in dermatological research due to its close resemblance in terms of structure, lipid composition, and barrier function [28]; the use of pig skin as a model enables assessing the formulation’s potential for human applications, providing essential insights into its performance before advancing to clinical studies.

No CUR permeation through the skin was observed for either the nanomicellar liquid dispersion (TPGS30ELP15) or hydrogel formulation (TPGS30ELP15-HY). CUR was not detectable in the receiving phase during the 24 h of the experiment.

**Figure 8:**
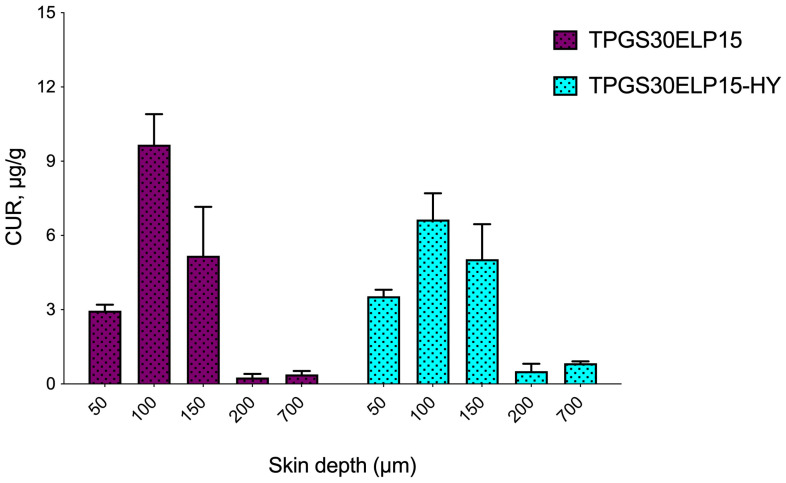
CUR retained (μg/g) into skin depth after the application of TPGS30ELP15 nanomicellar dispersion and TPGS30ELP15-HY formulations. Data are reported as mean ± SEM (n = 4).

The results of in vitro cutaneous penetration experiments are presented in the histogram (Figure 8), which depicts the amount of CUR retained in each skin layer (expressed in micrograms per gram of skin) across the full skin thickness (from 50 to 700 µm).

The nanomicellar dispersion (TPGS30ELP15) showed a higher accumulation of CUR at depths of 100 and 150 µm (9.66 ± 1.24 and 5.18 ± 1.97 μg/g, respectively) compared to the superficial 50 µm layer (2.96 ± 0.24 μg/g). This was followed by a sharp decline in the CUR level in deeper layers. This distribution pattern can be attributed to the smaller size of the nanomicellar formulation.

Several studies have demonstrated a correlation between smaller particle sizes, typically ranging from 12 to 25 nm, and the increased retention of active compounds within the skin [8,9,29]. This nanoscale particle size not only facilitates improved contact with the skin surface but also enables nanomicelles to bypass the stratum corneum (SC), the primary barrier to drug penetration. This characteristic is crucial for achieving effective drug concentrations at the target site while accounting for the skin’s inherent barrier properties.

Traditional topical formulations, such as creams, gels, and ointments, often fail to achieve sufficient drug penetration into deeper skin layers. In contrast, polymeric nanomicelles represent an innovative solution for enhanced drug delivery to the skin. Numerous studies have highlighted their ability to improve targeted drug delivery. Key advantages of polymeric micelles as skin nanocarriers include enhanced drug solubilization, increased partitioning of hydrophilic drugs into the SC, and localized drug deposition in hair follicles and keratinocytes across various epidermal layers.

Furthermore, polymeric micelles provide a depot effect, enabling slow and sustained drug release from intact micelles. Remarkably, even in cases of skin barrier disruption associated with dermatological disorders, polymeric micelles have demonstrated their efficiency as carriers for targeted drug delivery [9].

In our study, CUR has purely lipophilic characteristics (Log P = 2.4 [5]) with a presumably greater affinity for the SC. However, our findings show lower accumulation in the first analyzed layer (50 µm), which includes the SC [28,30]. This suggests that the nanomicellar structure plays a crucial role in facilitating the transport of the drug into deeper layers (100 and 150 µm), which are primarily composed of the viable epidermis [28,30]. These deeper layers, being more hydrophilic, would otherwise hinder the drug’s penetration, underscoring the importance of nanomicelles in overcoming such challenges.

When the nanomicellar dispersion was introduced into the thermosensitive hydrogel, the same trend was maintained with a peak in the accumulated CUR amount at 100 µm (6.64 ± 1.06 µg/g) of thickness exhibiting a more gradual decrease, maintaining measurable levels of CUR (0.83 ± 0.08 µg/g) even at a depth of 700 µm. Compared to TPGS30ELP15 nanomicellar dispersion, the hydrogel resulted in a more evenly distributed CUR amount in the upper skin layers (SC and viable epidermis). This suggests that the hydrogel provides a more balanced and effective drug penetration profile, potentially enhancing skin retention.

**Figure 9:**
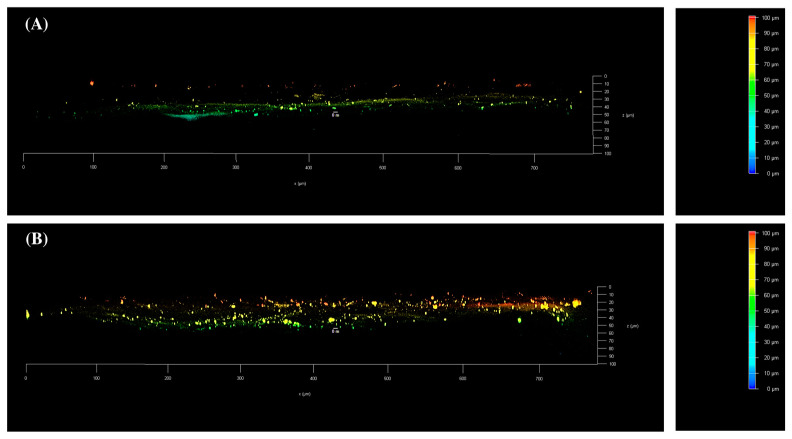
A 3D confocal reconstruction of skin samples with a draw depth coding scale bar. (**A**) Skin treated with the TPGS60ELP30 formulation; (**B**) skin treated with the TPGS60ELP30-HY formulation.

Following the 24 h permeation study, selected skin samples were analyzed using confocal laser scanning microscopy (CLSM). This technique, widely documented in the scientific literature [30], allows for detailed visualization of the fluorescent CUR’s penetration depth, providing valuable insights into its distribution and localization within the skin. The analysis of the skin samples indicates that the TPGS30ELP15 nanomicellar dispersion allowed the penetration of CUR into the skin. After 24 h following the application of the formulation, CUR was observed to penetrate beyond the superficial layers, concentrating at a depth of 40 to 60 μm (Figure 9A). A rapid release of the active ingredient was noted, followed by its diffusion into the underlying skin layers. In the case of the TPGS30ELP15-HY hydrogel, a uniform distribution of CUR was observed from the skin surface to a depth of 70 μm. Unlike the nanomicellar dispersion, no advancing front of CUR was detected. Instead, the homogeneous color distribution suggested a continuous release of the drug over time (Figure 9B). This behavior can be attributed to the rheological properties of the hydrogel, which, despite its viscosity potentially hindering diffusion, facilitated a homogeneous distribution of CUR over time. It is noteworthy that the microscopic analysis was conducted following 24 h of contact. It is hypothesized that an equilibrium state may be achieved after a certain penetration time [31], allowing the greater amount of CUR initially detected in the first layers treated with the thermosensitive gel to move into deeper layers once equilibrium is established. Additionally, the images reveal the presence of “bright spots” on the skin treated with the hydrogel, which may indicate a significant cutaneous deposition of CUR. For both formulations, no detectable levels of CUR were observed beyond a depth of 70 μm.

**Figure 10:**
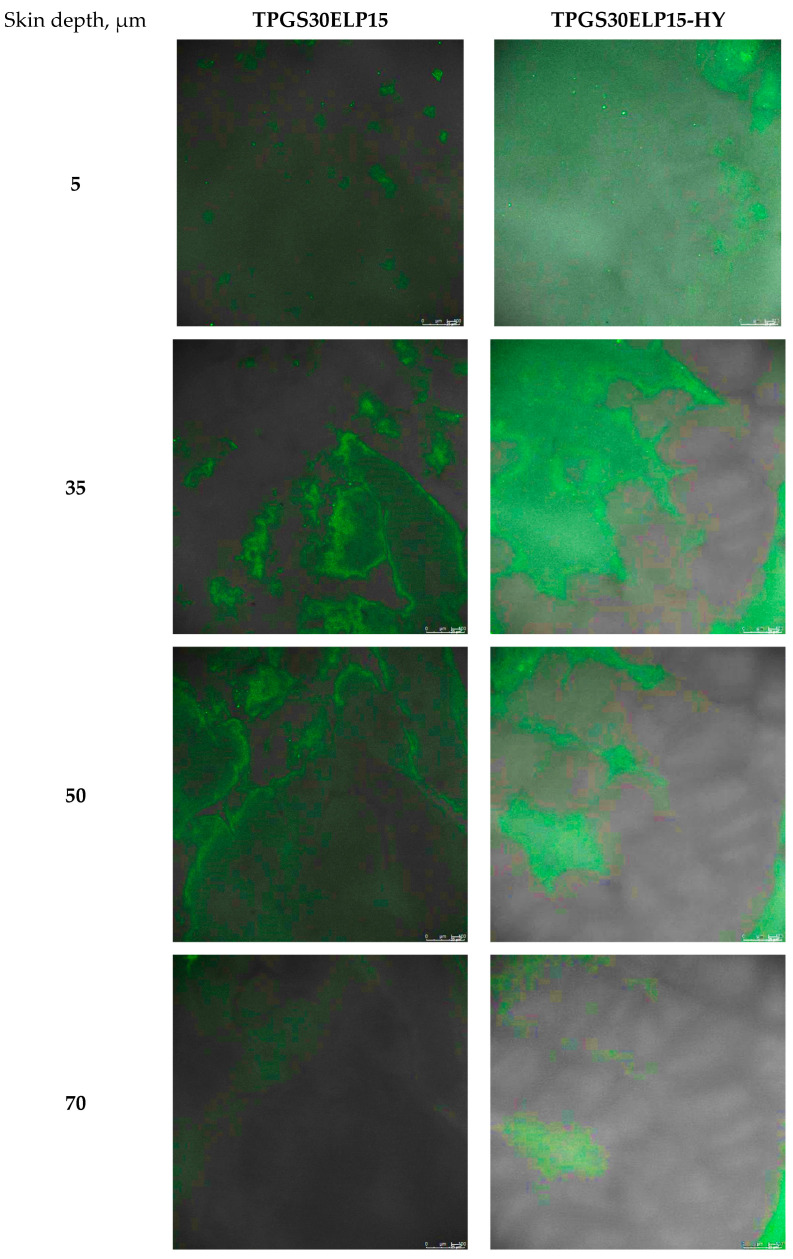
Z-stack images of the skin samples subsequent to the application of the nanomicellar formulation containing CUR in liquid form (TPGS30ELP15) or as a thermosensitive hydrogel (TPGS30ELP15-HY) obtained by scanning samples from the skin surface (z = 0 μm) down to deeper skin layers (z = 100 μm) with a z-step size of 1 μm.

CUR fluorescence was observed throughout the entire depth of the SC and into the viable epidermis, as shown in Figure 10, which presents representative z-stack images. This is consistent with the findings of Jacobi et al. [28], who reported that the viable epidermis of pig ear skin has a thickness of 60–85 μm, while the SC measures 17–28 μm.

The nanomicellar formulation, whether a colloidal dispersion or incorporated into a thermosensitive hydrogel, effectively facilitates the penetration of CUR into the outer skin layers, reaching the deeper regions of the vital epidermis. This distribution corresponds with the location of melanocytes, the primary target cells for melanoma therapy, highlighting the system’s efficacy in delivering CUR precisely to the intended site of action. Localized retention significantly reduces systemic toxicity and minimizes adverse effects, thereby enhancing the formulation’s safety profile. Moreover, the targeted delivery to the epidermis ensures that therapeutic concentrations of CUR are achieved at the tumor site while safeguarding surrounding tissues from unnecessary exposure. This targeted approach is essential for improving the efficacy and safety of topical drug delivery systems, especially for localized conditions, such as melanoma.

## 3. Materials and Methods

### 3.1. Materials

The following materials were used: curcumin (CUR, molecular weight 368.38 g/mol); polyethylene glycol sorbitan monooleate (Tween 80) and sodium dodecyl sulfate (SDS) purchased from Sigma-Aldrich, St. Louis, MO, USA; pharmaceutical-grade d-α-Tocopherol Polyethylene Glycol Succinate (Vitamin E-TPGS, TPGS 1000, molecular weight 1513 g/mol, PMC ISOCHEM, Vert-Le-Petit, France, TPGS); polyoxyl-35-castor oil (Kolliphor ELP, molecular weight 2500 g/mol, ELP); and poly(ethylene glycol)-*block*-poly(propylene glycol)-*block*-poly(ethylene glycol) (Kolliphor^®^ P407) from BASF, Ludwigshafen, Germany. All other chemicals and solvents were of analytical grade. Ultrapure water was prepared using the Milli-Q^®^plus apparatus (Millipore, Milan, Italy).

### 3.2. Preparation of CUR Nanomicelle-Loaded Thermosensitive Hydrogel (TPGS30ELP15-HY)

The nanomicelle-loaded thermosensitive hydrogel (TPGS30ELP15-HY) was prepared upon the addition of the freeze-dried nanomicellar formulation containing CUR (TPGS30ELP15-F) to a 16% Kolliphor^®^ P407 aqueous dispersion under continuous stirring for 12 h at 4 °C. TPGS30ELP15 formulation was prepared as reported by Paganini et al. [1]. Briefly, TPGS and ELP, used in a molar ratio of 30:15, were melted at 50 °C for 1 h. Subsequently, an excess of CUR (8.14 mM) was added, and the mixture was stirred until a homogeneous blend was obtained. After water addition, stirring for 12 h, and filtering through 0.20 µm pore size filters (0.20 μm RC Syringe filter, Phenomenex, Torrance, CA, USA), a clear nanomicellar dispersion was formed (TPGS30ELP15). Then, the formulation was freeze dried (TPGS30ELP15-F) in a VirTis apparatus (VirTis Wizard 2.0, SP Scientific, Gardiner, NY, USA) under the following operating conditions. *Freezing* had a pressure of 400 torr; a temperature of −38 °C; a rate of 0.6 °C/h; and an extra freeze time of 120 min. *Primary drying* had a pressure of 100 torr; a temperature from −38 to 0 °C at a rate of 2.1 °C/h. *Secondary drying* had a pressure of 50 torr; a temperature from 0 to 25 °C at a rate of 5.0 °C/h; and extra drying at 27 °C for 60 min.

### 3.3. Characterization of CUR Nanomicelle-Loaded Thermosensitive Hydrogel (TPGS30ELP15-HY)

The thermosensitive hydrogel loaded with CUR nanomicelles (TPGS30ELP15-HY) was evaluated to confirm the maintenance of the physicochemical properties of the CUR-loaded nanomicelles within the thermosensitive matrix, specifically in terms of particle size and encapsulation efficiency.

First of all, we ensured that the freeze-drying process did not induce any alterations to the liquid nanomicellar formulation by reconstituting the lyophilized product with the appropriate amount of water or with a 16% Kolliphor^®^ P407 aqueous dispersion, followed by agitation for 12 h at room temperature or at 4 °C, respectively. Nanomicelle size by dynamic light scattering, the amount of drug encapsulated by HPLC, and entrapment efficiency (EE%) were determined before and after lyophilization and on the final hydrogel (TPGS30ELP15-HY) as previously described by Paganini et al. [1].

To quantify the CUR amount into the different formulations, the samples were filtered, diluted with ethanol, sonicated, and centrifugated before the HPLC analysis.

Moreover, NMR analysis was carried out to verify if the nanomicelles retained their structural integrity in the presence of Kolliphor^®^ P407 and the loaded CUR.

Subsequently, the rheological behavior and the sol–gel transition at the skin surface temperature of TPGS30ELP15-HY hydrogel were investigated in depth through the tube inversion method [24] and rheological analysis.

#### 3.3.1. Dynamic Light Scattering Analysis

The average hydrodynamic diameter (Dh) and polydispersity index (PDI) of the nanomicellar formulations were determined by the Dynamic Light Scattering (DLS) technique using Zetasizer Nano ZS (Malvern). Just before the DLS measurements, each sample was appropriately diluted with ultrapure water freshly filtered through 0.20 µm pore size filters to ensure that the concentration of nanomicelles fell within the measurement intensity range of 5 × 10^4^ to 1 × 10^6^ counts per second. Dh and PI were measured at 25 °C with three runs for each sample using an angle of 173° and a run time of 200 s.

#### 3.3.2. NMR Analysis

NMR spectra of the samples of nanomicelles without CUR (TPGS30ELP15-empty), CUR-loaded formulation (TPGS30ELP15), Kolliphor^®^ P407, raw material, and CUR-loaded nanomicelles in the presence of Kolliphor^®^ P407 (TPGS30ELP15-HY) were measured on a JEOL JNM-ECZ500R spectrometer operating at 500 MHz for ^1^H (JEOL Italia S.p.A., Milan, Italy). All NMR spectra were referenced through the solvent lock (^2^H) signal according to the IUPAC recommended method and the manufacturer’s protocols. The solvent suppression sequence employed was WATERGATE with perfect echo. All deuterated solvents used were purchased from Deutero (Deuteto GmbH, Kastellaun, Germany). CUR samples were freshly prepared and immediately analyzed.

#### 3.3.3. Gel–Sol Transition Temperature Determination

As for the tube inversion method, two Eppendorf tubes, each containing 0.5 mL of the hydrogel, were first equilibrated at 4 °C. Afterward, they were immersed in a water bath set to 40 °C to observe the physical changes resulting from the temperature shift. A thermometer was placed in one tube to serve as the control, while the other tube was inverted every 3 min, starting from the up position, to assess the flow properties of the hydrogel. The temperature at which the solution ceased dropping was recorded as the gelation time. Measurements continued until the samples reached room temperature, after which they were cooled again to confirm the transition temperature through a second round of measurements.

Rheological measurements were performed using a rotational viscometer (Rheostress 1, Haake, Thermo Fisher Scientific, Karlsruhe, Germany) equipped with coaxial cylinders measuring geometries (Z41) and a thermostated system for temperature control. Data acquisition was managed through Rheowin software (version 4.61.0003, Haake Thermo Fisher Scientific, Karlsruhe, Germany). Rheograms were generated for temperature values ranging from 20 to 45 °C at a constant speed of 100 s^−1^ over a duration of 1000 s. The relationship between viscosity (μ) and temperature gradient (T) was analyzed by applying mathematical analysis to the experimental data points obtained from the rheograms. Stress sweep measurements were conducted from 0.1 to 10 Pa at a constant frequency (1 Hz) to define the range of linear viscoelasticity (LVR) and to assess the maximum deformation achievable by the sample. Subsequently, the temperature dependence of the dynamic moduli, G′ (storage modulus) and G″ (loss modulus), were determined by oscillation temperature sweeps from 20 ± 1 °C to 45 ± 1 °C with constant stress (5 Pa) within the LVR. The temperature at which the G′ and G″ curves intersect (cross-over temperature) is defined as the gelation temperature.

### 3.4. In Vitro CUR Release Studies

The in vitro release study of TPGS30ELP15, both in solution and hydrogel forms, was performed using the dynamic dialysis method. One milliliter of either the nanomicellar dispersion (TPGS30ELP15) or the thermosensitive hydrogel (TPGS30ELP15-HY) was placed in a closed dialysis bag (MWCO 6000 Da, Spectra/Pore 3 Dialysis Membranes, Spectrum Labs, Breda, The Netherlands). The dialysis bags were immersed in 20 mL of a receiving medium consisting of 1% Tween 80 solution, maintained at 32 °C to simulate skin surface temperature, and continuously stirred. To ensure sink conditions, 1.0 mL of the receiving medium was replaced with a fresh solvent at predetermined time intervals (4, 8, 24, 32, 48, 54, and 72 h). The experiment was carried out over a 48 h period, with each condition tested in triplicate. The drug concentration in the receptor medium was quantified using fluorescence analysis.

### 3.5. Skin Permeation/Penetration Study

#### 3.5.1. In Vitro Permeation Experiment

In vitro skin permeation studies were performed through porcine ear skin after topical application of TPGS30ELP15 or TPGS30ELP15-HY. The experimental procedure followed the method described by Tampucci et al. [32], utilizing Gummer-type diffusion cells. Porcine ear skin, obtained from freshly sacrificed animals at a local slaughterhouse, was used as a model. After rinsing with water, full-thickness skin was carefully excised from the outer cartilage region using a scalpel, and any adhering fat and subcutaneous tissues were removed. Before starting the experiments, the hair was trimmed and the skin was gently washed. Skin samples were placed in the Gummer-type diffusion cells with a diffusion area of 1.23 cm^2^. The donor compartment was filled with 1 mL of TPGS30ELP15 or TPGS30ELP15-HY formulation, while the receiving compartment contained 5 mL of deionized water, maintained at 37 °C, and stirred at 600 rpm. At predetermined time intervals, 5 mL samples of the receiving phase were withdrawn and replaced with the same volume of fresh fluid to ensure the sink conditions. A total of 500 μL of each sample was vacuum dried, reconstituted in 100 μL of ethanol, and analyzed by HPLC. All experiments lasted 24 h and were replicated four times.

#### 3.5.2. Skin Cryo-Sectioning Experiment

At the end of the permeation experiments, the skin was collected, rinsed with distilled water to eliminate excess vehicles from the skin surface, and gently wiped with cotton wool tampons. The samples were then frozen and sectioned horizontally into 50 μm thick slices with a cryomicrotome (MEV Cryostat, Slee-Technik GMBH, Mainz, Germany) following the procedure described by Monti et al. [33]. CUR extraction from the skin sections was performed by treatment with 1.0 mL of 2% SDS solution for 24 h. After contact with 2.0 mL of methanol for 1 h, the mixture was centrifuged at 4000 rpm for 15 min. A 100 μL aliquot of the supernatant was vacuum dried and reconstituted in ethanol for HPLC analysis. To validate the extraction procedure, untreated skin samples (blank) were analyzed, and the retention time of endogenous compounds was compared with that of CUR to confirm the absence of analytical interferences. Additionally, a known quantity of CUR was spiked into blank skin samples, and the extraction recovery was determined by calculating the ratio of CUR extracted to the initial quantity added. The recovery was in the range of 88–95%.

### 3.6. Analytical Method

The quantitative determination of solubilized CUR in the formulations under study (TPGS30ELP15, TPGS30ELP15-F, and TPGS30ELP15-HY) in the receiving phase and in the skin was carried out by HPLC analysis. The HPLC system included a Shimadzu LC-20AD unit equipped with a UV-Vis SPD-10A detector, an SIL-10AD VP automatic injector (20 μL), and a C-R4A integrator (Shimadzu Italia s.r.l., Milan, Italy). A Rheodyne injection valve with a 20 μL capacity was used. Chromatographic separation was achieved with a Luna-C8 column (10 μm; 250 × 4.6 mm, Phenomenex, Torrance, CA, USA), which was thermostated at 30 °C using a Thermasphere™ column temperature controller (Phenomenex, Torrance, CA, USA). The mobile phase (flow rate of 1.0 mL/min) consisted of acetonitrile/acetic acid 4% *v/v* in a 55:45 ratio. Detection was performed at a wavelength of 424 nm, with a retention time of approximately 5 min.

CUR amount in the samples was determined by comparison with the external standard curve obtained by adding increasing amounts of the product to ethanol after checking that there were no interferences from the matrix under study (nanomicelles, receiving phase, skin). The standard curve was linear within the concentration range of 0.0526–1.052 µg/mL (R^2^ = 0.00986) with limits of quantification (LOQ) and determination (LOD) at 0.05 and 0.017 µg/mL, respectively.

In the case of in vitro release studies, the released CUR amount was quantified by fluorescence analysis using a multimode microplate reader (Varioskan^TM^ Luxs/n 3020-815, Thermo Scientific^TM^, Milan, Italy). The relative fluorescence intensity of the samples was measured at 538 nm emission after excitation at 485 nm. The results were obtained based on an external calibration curve, and the standard curve showed linearity in the concentration range of 0.0597 and 22.96 μg/mL (y = 1.3751x + 1.6936, r^2^ = 0.9959).

### 3.7. Visualization of CUR Skin Penetration Using Confocal Laser Scanning Microscopy (CSLM)

Following the permeation study, skin samples were analyzed using a Leica TCS SP8 laser scanning confocal microscope (Leica Microsystems, Wetzlar, Germany) equipped with a 20× objective lens (HC PL APO CS2 20x/0.75 DRY) and a zoom factor of 0.75. An argon laser with an excitation wavelength of 488 nm was employed to stimulate CUR fluorescence [34,35]. This wavelength is sufficiently distinct from the excitation range of endogenous porcine skin fluorophores, such as collagen, elastin, and aromatic amino acids, which typically exhibit excitation maxima between 295 nm and 360 nm. This separation minimizes interference from skin autofluorescence, allowing for more precise visualization of CUR distribution. A hybrid detector (HyD) and transmission photomultiplier tubes (PMTs) were employed to capture CUR’s green fluorescence signals and to emphasize the morphological characteristics of skin tissues, respectively. Three-dimensional (3D) reconstruction of the skin samples was achieved using the xyz scan mode, in which two-dimensional (2D) confocal cross-sectional images were sequentially captured along the xy and xz planes. This approach enabled the acquisition of multiple cross-sectional images spanning the full thickness of the tissue.

In particular, z-stacks were obtained by scanning samples from the skin surface (z = 0 μm) down to deeper skin layers (z = 100 μm) with a z-step size of 1 μm. To visualize the CUR fluorescence intensity brightness expression, the Maximum Intensity Projection (MIP) function was applied, combining each pixel from multiple cross-sectional images into a single representative image. Three-dimensional confocal z-stack images of the skin samples, with a depth-coded scale bar, were generated to track the fluorescent signal, indicating CUR permeation through deeper skin layers. The images were reorganized and processed using Leica Application Suite (LAS) X software, version 3.5.5.19976 (Leica Microsystems, Wetzlar, Germany).

### 3.8. Statistical Analysis

All data are presented as means ± standard errors. Statistical differences were evaluated for two groups applying Student’s two-tailed unpaired *t*-test (GraphPad Prism Software, version 10, San Diego, CA, USA).

Statistical analyses for multiple groups were determined by the one-way ANOVA test and Dunnett’s test for multiple comparisons. Data were considered statistically significant with *p*-values of * <0.05, ** <0.01, *** <0.001, and **** <0.0001.

## 4. Conclusions

This study underscores the significant potential of a nanomicellar-based delivery system, particularly when integrated into a thermosensitive hydrogel, for enhancing the skin penetration and retention of curcumin (CUR). The binary surfactant system, composed of TPGS and ELP, successfully formed stable nanomicelles (TPGS30ELP15) with high CUR solubilization capacity, leading to a pronounced reduction in melanoma cell viability. Incorporating this nanomicellar formulation into a thermosensitive hydrogel (TPGS30ELP15-HY) not only preserved the physicochemical stability of the nanomicelles but also facilitated targeted delivery to the skin layers, as demonstrated by in vitro release studies and skin penetration assessments.

The nanomicellar dispersion enabled CUR to penetrate deeper layers of the epidermis, whereas the hydrogel ensured a more uniform drug distribution within the skin. This behavior is particularly advantageous for topical applications in melanoma therapy, where precise and localized delivery to melanocytes is of paramount importance. Additionally, the thermosensitive hydrogel prolonged skin contact and improved bioavailability, further enhancing therapeutic efficacy while promoting better patient compliance.

Overall, the combination of an in situ gelling hydrogel with a nanomicellar delivery system represents a highly promising approach for topical CUR delivery in skin cancer treatment. Its ability to enhance drug solubilization, maintain prolonged skin contact, and achieve targeted drug localization highlights its potential for further development and therapeutic applications.

## Figures and Tables

**Table 1:** The influence of the various stages involved in the preparation of the final hydrogel on particle size (Dh, PDI), solubilized CUR content, and encapsulation efficiency (EE) (mean ± SE, n = 3).

Formulation	D_h,_ nm	PDI	CUR, mM	EE, %
TPGS30ELP15	13.11 ± 0.01	0.371 ± 0.05	5.51 ± 1.09	67.72 ± 13.46
TPGS30ELP15-F	15.67 ± 0.33	0.170 ± 0.01	4.90 ± 0.88	60.20 ± 10.81
TPGS30ELP15-HY	17.97 ± 0.39	0.167 ± 0.01	4.72 ± 0.23	57.98 ± 2.83

**Table 2:** Release rate (µg/h) and percentage of CUR released at 48 h (Q_48h_) and 72 h (Q_72h_) for TPGS30ELP15 and TPGS30ELP15-HY (mean ± SE, n = 3).

Formulation	Release Rate	Q_48h_	Q_72h_
TPGS30ELP15	4.80 ± 0.71	11.38 ± 1.95	11.12 ± 0.46
TPGS30ELP15-HY	2.09 ± 0.29	5.81 ± 0.37	7.25 ± 1.07

## Data Availability

The original contributions presented in this study are included in the article. Further inquiries can be directed to the corresponding author.
